# Treatment with onion bulb extract both prevents and reverses allergic inflammation in a murine model of asthma

**DOI:** 10.1080/13880209.2024.2335187

**Published:** 2024-04-08

**Authors:** Ahmed Z. El-Hashim, Maitham A. Khajah, Khaled Y. Orabi, Sowmya Balakrishnan, Hanan G. Sary, Ahmad M. Barakat

**Affiliations:** a Department of Pharmacology & Therapeutics, Faculty of Pharmacy, Kuwait University, Kuwait City, Kuwait; b Department of Pharmaceutical Chemistry, Faculty of Pharmacy, Kuwait University, Kuwait City, Kuwait

**Keywords:** House dust mite, signaling pathways, EGFR, AKT, erk1/2, a, nd IgE

## Abstract

**Context:**

Asthma presents a global health challenge. The main pharmacotherapy is synthetic chemicals and biological-based drugs that are costly, and have significant side effects. In contrast, use of natural products, such as onion (*Allium cepa* L., Amaryllidaceae) in the treatment of airway diseases has increased world-wide because of their perceived efficacy and little safety concerns. However, their pharmacological actions remain largely uncharacterized.

**Objective:**

We investigated whether onion bulb extract (OBE) can (1) reverse established asthma phenotype (therapeutic treatment) and/or (2) prevent the development of the asthma phenotype, if given before the immunization process (preventative treatment).

**Materials and methods:**

Six groups of male Balb/c mice were established for the therapeutic (21 days) and five groups for the preventative (19 days) treatment protocols; including PBS and house dust mite (HDM)-challenged mice treated with vehicle or OBE (30, 60, and 100 mg/kg/i.p.). Airways inflammation was determined using cytology, histology, immunofluorescence, Western blot, and serum IgE.

**Results:**

Therapeutic (60 mg/kg/i.p.) and preventative (100 mg/kg/i.p.) OBE treatment resulted in down-regulation of HDM-induced airway cellular influx, histopathological changes and the increase in expression of pro-inflammatory signaling pathway EGFR, ERK1/2, AKT, pro-inflammatory cytokines and serum IgE.

**Discussion and Conclusion:**

Our data show that OBE is an effective anti-inflammatory agent with both therapeutic and preventative anti-asthma effects. These findings imply that onion/OBE may be used as an adjunct therapeutic agent in established asthma and/or to prevent development of allergic asthma. However, further studies to identify the active constituents, and demonstrate proof-of-concept in humans are needed.

## Introduction

Asthma is a chronic inflammatory disease that leads to airway remodeling, and current estimates show that over a third of a billion people are affected by this disease globally (Kuruvilla et al. 2019). Whilst new biologic antibody-based therapy has been introduced over the last few years, targeting mainly asthmatics with severe symptoms, the mainstay treatment still remains steroid-based anti-inflammatory and long-acting bronchodilators (O’Byrne et al. 2001; Williams 2018; Zhang Y et al. 2018; Global Initiative For Asthma [GINA] 2019). A major drawback of the current asthma medicines is that not only are they beyond the financial reach of tens of millions of people world-wide, but their use is associated with a high side effect profile (Harrison et al. 2015; Agache et al. 2020). Importantly, a significant percentage of asthmatic patients achieve only sub-optimal therapeutic outcomes (Pinto et al. 2013). Hence, effective asthma treatment remains an unmet global challenge.

The important driving factors for asthma pathogenesis, and those probably shaping the asthma trajectory, are a combination of genetic predisposition, environmental factors and the environmental microbiome, particularly allergens (Yang et al. 2017). The net result of this complex series of interactions between different components of the environment, in the context of genetic susceptibility, are changes in both the epigenome and transcriptome that eventually lead to activation of Th2 cell thus giving rise to an allergic phenotype (Yang et al. 2017). Any intervention within this cycle may result in a change in outcome. One obvious avenue for intervention is *via* diet. Studies have shown that an intake of a balanced diet that has an abundance in sources of antioxidants (e.g., fruits and vegetables), such as the Mediterranean diet, may be advantageous in the prevention of asthma.

The use of natural products, particularly herbs such as onions, for medicinal purposes has been on the rise globally (Newman and Cragg 2016). A large number of asthmatic patients in the developing world, and also in the developed world, use natural products either as their main supplementary nutrition or as anti-inflammatory therapy (Clark et al. 2010). *Allium cepa* L. family) is a very popular plant which is used in the management of numerous ailments including that of airway dysfunction such as cough, asthma and bronchitis (Lanzotti 2006; Toh et al. 2013; Gormaz et al. 2015; Oliveira et al. 2015; Upadhyay 2016; El-Hashim et al. 2017; Tang et al. 2017; Zeng et al. 2017). We have recently shown that OBE has effective anti-inflammatory effects in animal models of asthma and colitis (Khajah et al. 2019; El-Hashim et al. 2020), and also reverses established bowel inflammation (Khajah et al. 2020). In the allergic asthma model, our findings showed that OBE, administered 1 h prior to allergen challenge, significantly inhibited the allergen-induced increase in airway cellularity and histopathological changes, and also pro-inflammatory Th2 cytokines such as IL-4 and IL-5 but interestingly enhanced the levels of the anti-inflammatory cytokine IL-10 (El-Hashim et al. 2020). We also demonstrated that OBE inhibited the EGFR-dependent signaling pathways (El-Hashim et al. 2020), which were reported to be important in both clinical and preclinical studies (Takeyama et al. 2001; El-Hashim et al. 2017). Hence, there is good evidence that onion/OBE has anti-inflammatory action, particularly when administered immediately before the inflammatory inducing agent.

Studies evaluating drug efficacy for “proof of concept” for a role in asthma disease pathogenesis have classically used the “disease prophylaxis approach”, which is where the drug is administered just before allergen challenge (El-Hashim et al. 2020). However, whilst many of these studies have demonstrated preclinical efficacy for a particular drug, this has not always been translated to clinical efficacy (Martin et al. 2014). Therefore, administering a drug prior to disease induction may very well inhibit the disease phentotype (Blease et al. 2001); however, this does not mean that it will reverse already established disease features. Therefore, the timing of when a drug is given, in preclinical studies, is critical to whether the findings of the studies are translatable to humans and to the success of the therapy.

What has not yet been established is whether onion/OBE treatment can reverse established airway inflammation and/or inhibit the development of the allergic status/allergic airway inflammation if administered prior to allergic sensitization. In this study, using an allergic murine asthma model, with several similarities to human asthma (Epstein 2006), we investigated 1) whether OBE can reverse established asthma phenotype (therapeutic treatment approach), 2) whether OBE can prevent the development of the asthma phenotype if given before the immunization process (preventative treatment approach), and 3) the mechanisms by which this may be achieved.

## Materials and methods

### Acquisition of plant material

Locally purchased (Kuwait vegetable market in February 2017 and March 2018) fresh red onion bulbs were identified as *Allium cepa* (by Thomas Mathew, PhD, Kuwait University Herbarium, Department of Biological Sciences, Kuwait University). The onion bulbs were assigned a specimen ticket number KOE-010 and deposited at the herbarium of Kuwait University (KTUH), College of Science, Kuwait.

### Extraction of onion bulbs

Approximately 40 kg of red onion was peeled and coarsely cut and percolated three times, each using 20 L of dichloromethane. The dichloromethane layers of the percolates were separated from the aqueous layer, dried over anhydrous sodium sulphate, and then evaporated in *vacuo* untill dryness to obtain a brownish syrupy residue. This extraction process was repeated as needed. The treatment stock solution was prepared using the residue and PBS as a vehicle.

Table 1 lists the compounds identified in *Allium cepa* bulb extract (El-Hashim et al. 2020)

**Table 1.** Compounds identified in *Allium cepa* bulb extract.

Identified compounds^a^	Retention time t_r_ (min)	Area (%)
Dipropyl disulfide	8.56	45.93
Dipropyl trisulfide	13.64	3.16
3,5-Diethyl-1,2,4-trithiolane	13.96	1.88
Propylpropane thiosulfonate	15.09	5.07
1,5-Dithiaspiro[5.6]dodecan-7-ol	20.32	3.46
Methyl-2,6-anhydro-3,4,7-tridesoxy-1-erythro-hept-2-enulonate	22.85	1.25
Tricosane	30.99	0.63
Pentacosane	33.74	1.37
1,2-Benzenedicarboxylic acid	34.06	0.34
Hexacosane	35.06	0.3
1-Heptacosanol	35.99	0.18
Nonacosane	36.34	1.2
Nonacosane	39.67	1.88
Tetratriacontane	45.23	4.09

^a^Identified by matching with mass spectral data in the NIST MS Search 2.0 library stored in the GC-MS system, and comparison with data reported in the literature (Taken with permission from El-Hashim et al. 2020).

### Animals

Male BALB/c mice (6–8 weeks old, average weight 25 g) were utilized in this study in accordance with the ARRIVE guidelines for reporting animal experiments. All the animal experimental protocols were approved by the Ethical Committee for the Use of Laboratory Animal in Teaching and Research, Health Sciences Center, (P11613PT01-12/16), Kuwait University and followed the guidelines set by EU Directive 2010/63/EU and the National Institutes of Health Guide for the Care and Use of Laboratory Animals (NIH Publications revised 2011). All animals were maintained under similar conditions consisting of temperature-controlled (22–24 °C) habitat, artificial 12 h light/dark cycle and were given standard chow with water *ad libitum.*

### Immunization and intranasal challenge and drug treatment protocols

#### Protocol for therapeutic treatment experiments

**Figure 1.**
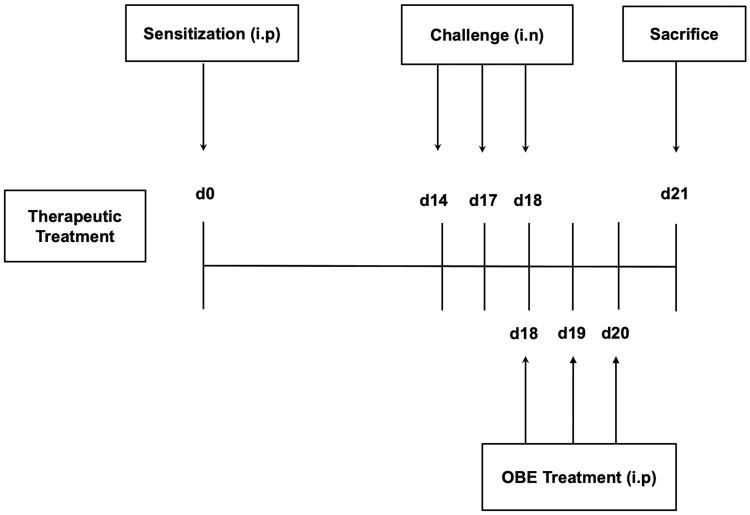
Schematic diagram illustrating the therapeutic protocol for HDM/PBS challenge and OBE treatment. Mice were immunized, intraperitoneally (i.p.) on day 0 and subsequently challenged intranasally (i.n.) on days 14, 17, and 18. Treatment with OBE (i.p.) was initiated on day 18 until day 20. Mice were sacrificed on day 21.

The immunization/challenge protocol have been previously described (El-Hashim et al. 2020). Six treatment groups (A–F, 7–11 mice per group) were designed to ascertain if OBE treatment would reverse the HDM-induced asthma phenotype. On day 0, all treatment groups were immunized with a single dose of 40 µg HDM in 0.2 mL of alu-Gel-S (Alu-Gel-S; SERVA Electrophoresis GmbH) by intraperitoneal (i.p.) injection. On days 14, 17, and 18, mice were challenged intranasally (i.n.) with PBS for group A (control group) and HDM for groups B-F. On day 18, groups A (*n* = 7) (PBS) and B (*n* = 11) (HDM/VEH) were treated (i.p.) with 0.2 mL of the drug vehicle (PBS). Groups C (*n* = 7), D (*n* = 7) and E (*n* = 9) were treated with 0.2 mL of OBE at doses 30, 60, and 100 mg/kg by i.p. injection, respectively. Additionally, group F (*n* = 9) received dexamethasone (DEX) (3 mg/kg/i.p.). All OBE/DEX treatment was done 6 h after the intranasal challenge. On day 19 and 20, groups A - F treatment was continued, however the PBS and HDM intranasal challenge was stopped. On day 21, mice were sacrificed with an overdose of halothane and bronchoalveolar lavage (BAL) was performed to obtain BAL fluid. Subsequently, lungs were excised and processed for histological samples and other experiments (Figure 1).

### Protocol for preventative treatment experiments

**Figure 2.**
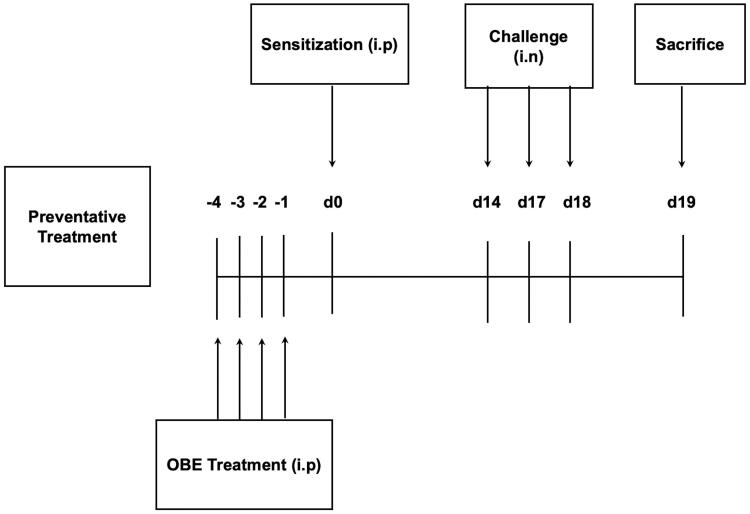
Schematic diagram illustrating the preventative protocol for HDM/PBS challenge and OBE treatment. Mice were immunized, intraperitoneally (i.p.) on day 0 and subsequently challenged intranasally (i.n.) on days 14, 17, and 18. Treatment with OBE (i.p.) was initiated 4 days prior to immunization and continued for four days. Mice were sacrificedon day 19.

Five treatment groups (A -E, *n* = 11 − 15 mice per group) were designed to ascertain if OBE treatment, given before HDM immunization, would prevent or inhibit the development of the HDM-induced asthma phenotype. Thus, 4 days before immunization with HDM, groups A (*n* = 14) (PBS) and B (*n* = 14) (HDM/VEH) were pretreated (i.p.) with 0.2 mL of the drug vehicle (PBS). Simultaneously, groups C (*n* = 11), D (*n* = 15) and group E (*n* = 15) were pretreated by (i.p.) with OBE doses 60 mg/kg, 100 mg/kg and DEX (3 mg/kg), respectively. On day 0, all treatment groups were immunized with a single dose of 40 µg HDM in 0.2 mL of alu-Gel-S (Alu-Gel-S; SERVA Electrophoresis GmbH) (i.p). On days 14, 17, and 18, group A (control) was challenged intranasally (i.n.) with 50 µL of PBS, whereas groups B-E were challenged (i.n.) with HDM in 50 µL of PBS. On day 19, mice were sacrificed with an overdose of halothane and BAL was performed to obtain BAL fluid. Furthermore, lungs were removed and processed for histological studies and other experiments (Figure 2). The mortality rate for mice during this study was about 10–15% and was mainly due to anesthesia, overdose with halothane, during the nasal allergen challenge.

For the histology and IF studies, OBE doses of 60 and 100 mg/kg were selected for the therapeutic and preventative treatment approaches, respectively, as they gave an optimal effects for those protocols.

### Bronchoalveolar lavage (BAL) fluid cell counts and lung histology

Mice were sacrificed by an overdose of halothane delivered by placing them in a glass jar with a piece of cotton soaked with 1 mL of halothane, thereafter BAL fluid was collected by cannulating the trachea and washing the lungs with saline solution (0.3 mL × 4 each). In brief, the number of BAL cells was calculated using a particle-size counter (Z1 Single Threshold; Beckman Coulter) then, cytosmears were prepared and cells were stained with Diff-Quik, afterwards, a differential count of 200 cells was performed using standard morphologic criteria. The total cell count reflects the number of cells per mL of BAL fluid. The differential cell count represents the absolute number for each cell type/mL of BAL fluid. For histological assessment, segments of lung tissue were excised and fixed in 10% buffered formalin, embedded in paraffin wax and sectioned into 5 µm thick slices. The sections were processed and stained separately with H&E stain and periodic acid–Schiff (PAS). Sections stained with PAS were later stained with Schiff, then with Mayer′s alum hematoxylin solution which caused bluing to occur. Sections were examined with a light microscope and the severity of histopathologic alterations was scored using a semi-quantitative 5-level lung histopathology score was used to assess the degree of inflammation in each microscopic field at 20× according to standard methods and analyzed and scored as previously described (El-Hashim et al. 2020). The scoring for H&E and PAS is semi-quantitative scoring system that considers both the staining intensity and cellularity. The score coding was as follows: (1 = normal, 2 = mild, 3 = moderate, 4 = severe, and 5 = highly severe). In brief, each slide is divided into four quadrants, and each quadrant is evaluated and given a score. The score for each slide/animal is then obtained by averaging all scores obtained. This process was done by two independent observers, blinded to the slides, and the final score given to each slide/animal was the average for two observers.

### Immunofluorescence

Lung tissues were processed as described above. Lung sections were incubated in a blocking solution (5% bovine serum albumin (BSA) + 0.3% Tween-20 in TBST) for 1 h and were subsequently incubated overnight at 4 °C with primary antibodies [p-EGFR (Tyr1068) (Rabbit; Cat. No. 3777S), pAKT (Ser 473) (Rabbit; Cat. No. 4060S) and pERK1/2 (Thr202/Tyr204) (Rabbit; Cat. No. 9101 L) (1:25 − 1:800 dilution) or only 1% BSA (for negative control); Cell Signaling, USA] diluted in 1% blocking solution. After 24 h, sections were washed and incubated with secondary antibody conjugated to Alexa Fluor 555 [Goat anti-rabbit IgG (Goat; Cat. No. 4413S) (1:1000 dilution); Cell Signaling, USA] for 2 h at room temperature in the dark. Following several washes in PBS, sections were stained with 4′, 6 diamidino-2-phenylindole (DAPI) and mounted. Images were captured with a 20× magnification on a ZEISS LSM 700 confocal microscope the laser setting and photo processing were equal among the different treatment groups for each protein. The fluorescence intensity was estimated in defined fields using the Image J software and equally modified in terms of sharpness and contrast to show the localization of the phospho-proteins in the lung tissue.

### Western blotting

Lobes from the dissected lungs of the mice were snap-frozen in liquid nitrogen and stored at −80 °C. Western blot was performed as described previously (El-Hashim et al. 2020). Briefly, samples were homogenized in lysis buffer and were allowed to lyse completely by incubation on ice for 30 min. The lysates were then centrifuged at 13,000 rpm for 10 min at 4 °C and the supernatants were collected, and the protein concentrations were estimated by Bio-Rad Bradford Protein Assay (Bio-Rad, Hercules, CA, USA). Aliquots with equal amounts of protein concentration (6 µg) were subjected to SDS-PAGE and transferred onto nitrocellulose membrane (Schleicher & Schuell, Dassel, Germany). The membranes were blocked with 5% BSA and then incubated with 1:1000 dilution; ERK1/2 (137F5) (Rabbit; Cat. No. 4695S), pERK1/2 (Thr202/Tyr204) (Rabbit; Cat. No. 9101L), Akt (Rabbit; Cat. No. 9272S), pAKT (Ser 473) (Rabbit; Cat. No. 9271S) and β-actin antibody (used as loading control, 1:1000 in 5% BSA) (Cell Signaling Technology, Boston, MA, USA) at 4 °C overnight. Membranes were incubated with appropriate secondary antibodies conjugated to horseradish peroxidase (Amersham, Buckinghamshire, UK) to detect phosphorylated form of ERK1/2 (42/44 kDa), Akt (60 kDa) or total form of actin (45 kDa). The immunoreactive bands were detected with Super Signal Chemiluminescent Substrate (Immuno Cruz Western blotting luminal reagent SC-20428, Santa Cruz Biotechnology) utilizing a Kodak autoradiography film (Care stream Biomax Xarfil 1660760). Images were analyzed and quantified generating data that was normalized to β-actin levels. Western blot experiments for each protein were run twice with lung samples from three different mice, in each treatment group (pooled), in each run.

### Measurement of lung cytokines

Mice lung tissues which were stored at −80 °C had the total protein concentration determined by Bradford analysis using the Bio-Rad Protein Assay reagent. Proteome Profiler™ Mouse Cytokine Array Kit (Catalog # ARY006, R&D Systems, Inc., MN, USA) was used to determine the relative variations of different cytokines and chemokines levels (IL1a, IL1b, IL5, IL16, IL10, GM-CSF, eotaxin, RANTES, MIP1a, MIP1b and MCP-5). The procedure was done according to the manufacturer’s protocol and previously described (Khajah et al. 2019). The experiment was run at least twice, with lung samples obtained from three different mice (pooled) for each treatment group, for each run.

### HDM-specific IgE levels in sera

HDM-specific IgE was detected using Chondrex MOUSE Serum Anti-HDM IgE Antibody Assay Kit, designed for the quantitative measurement of mouse anti-house dust mite IgE antibodies (Catalog #3037, Chondrex, Inc. Redmond, WA, USA). Cardiopuncture was used to collect mouse blood. Following anesthetizing the mice with halothane, an incision was made in the chest and a 24/25-gauge needle, attached to a syringe, was introduced into the heart through the left ventricle. Approximately, 0.3–0.5 mL of blood was collected and added to an Eppendorf tube with no additives and was then left standing for 15–20 min in order that the blood clots completely. These tubes were centrifuged at 1300 rpm for 5 min at 4 °C and the serum was removed and stored at −80 °C. Subsequently, the serum was then diluted with a dilution buffer (1:10 HDM-specific IgE), provided by the Chondrex MOUSE Serum Anti-HDM IgE Antibody Assay Kit. The assay was carried out according to the instructions of the manufacturer; 100 µL diluted standards (supplied in the kit) and samples were added into the wells of the pre-coated plate respectively which were then incubated at room temperature for 2 h. The plate was washed with buffer, then 100 µL of diluted biotinylated HDM was added into the wells and incubated at room temperature for 2 h. Next, the plate was washed with buffer and 100 µL of diluted Streptavidin Peroxidase was added to the wells, then incubated at room temperature for 30 min. The plate was washed with wash buffer and 100 µL of TMB solution was added into the wells then incubated at room temperature for 25 min. Finally, 50 µL of stop solution was added to the wells and the plate was read at 450 nm with a reference filter of 630 nm. IgE was determined by examining the OD (absorbance) values of our samples and the standards provided by the kit. This was then evaluated by producing a standard curve from the standard results obtained using the statistical software GraphPad Prism 6. This allowed us to determine the IgE concentration in our samples. This experiment was performed with more than 6 animals in each treatment group.

### Statistical analysis

Numerical values were expressed as means ± SEM. The data generated from therapeutic and the preventative studies concerning total and differential cell count, histopathological, immunofluorescence, cytokines and IgE were assessed for normality. Normally distributed data were assessed by one-way analysis of variance (ANOVA) followed by Bonferroni *post hoc* test (H&E and cytokines). Non-parametric data were analyzed by Kruskal-Wallis test followed by Dunn’s multiple comparison test followed by Dunn’s multiple comparison test (total count cells, PAS, immunofluorescence and IgE). The difference was considered as significant at a *P* value less than 0.05. All analyses were performed using GraphPad Prism 6 (CA, USA).

## Results

### Extraction of onion bulbs

The onion extraction process was carried out and replicated four times to produce 7.5 g (0.038% yield) of brownish syrupy residue. The dichloromethane extract of onion was previously analyzed using GC-MS to establish the identity of the plant and the constituents (El-Hashim et al. 2020). Generated mass spectrometry data lists the identified constituents in Table 1 (El-Hashim et al. 2020). The major sulfur-containing compounds detected included: dipropyl disulfide, dipropyl trisulfide, and propylpropane thiosulfonate, 3,5-diethyly-1,2,4-trithiolane. Other sulfur compounds were found, albeit at lower amounts.

### Effect of therapeutic OBE treatment on HDM- induced inflammatory cell infiltration

**Figure 3.**
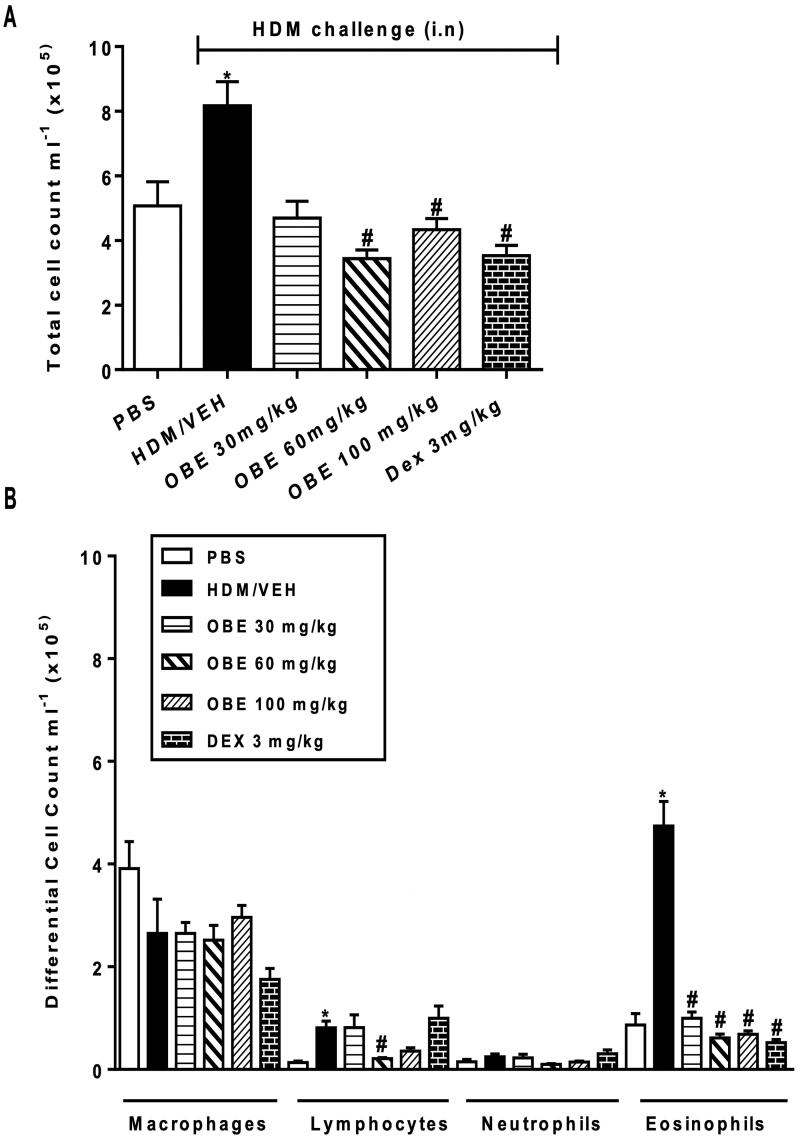
Effect of therapeutic OBE treatment (at dose 30, 60, and 100 mg/kg; i.p) on HDM-induced increase in (A) total cell and (B) differential cell count. Data are expressed as mean ± SEM (*n* = 7–11) *p < 0.05 vs. PBS and ^#^*p* < 0.05 vs. HDM (Kruskal-Wallis test followed by Dunn’s multiple comparison test).

HDM-challenged mice (VEH group) demonstrated significantly elevated counts in total cell (8.2 ± 0.7 × 10^5^ cells/mL), lymphocyte (0.8 ± 0.1 × 10^5^ cells/mL) and eosinophil numbers (4.7 ± 0.5 × 10^5^ cells/mL) in comparison with the control group (5.1 ± 0.7, 0.13 ± 0.03, 0.9 ± 0.2 × 10^5^ cells/mL, respectively, *p* < 0.05; Figure 3A, B, *n* = 7–11). However, this HDM-induced elevated total cell count observed in this group was significantly reversed following therapeutic OBE treatment, at either the 60 or 100 mg/kg dose (3.4 ± 0.3, 4.3 ± 0.3 vs. 8.2 ± 0.74 × 10^5 ^cells/mL; *p* < 0.05; Figure 3A, B, *n* = 7–11). Furthermore, OBE treatment reversed the rise observed in the HDM group in lymphocytes (0.2 ± 0.02 vs. 0.8 ± 0.1 × 10^5^ cells/mL, *p* < 0.05 at 60 mg/kg), eosinophils (1.0 ± 0.1, 0.6 ± 0.1, 0.7 ± 0.1 vs. 4.7 ± 0.5 × 10^5^ cells/mL, *p* < 0.05 at 30, 60 and 100 mg/kg, respectively), thus producing similar effects to those seen in the DEX group (Figure 3B).

### Effect of therapeutic OBE treatment on HDM-induced histopathological changes

**Figure 4.**
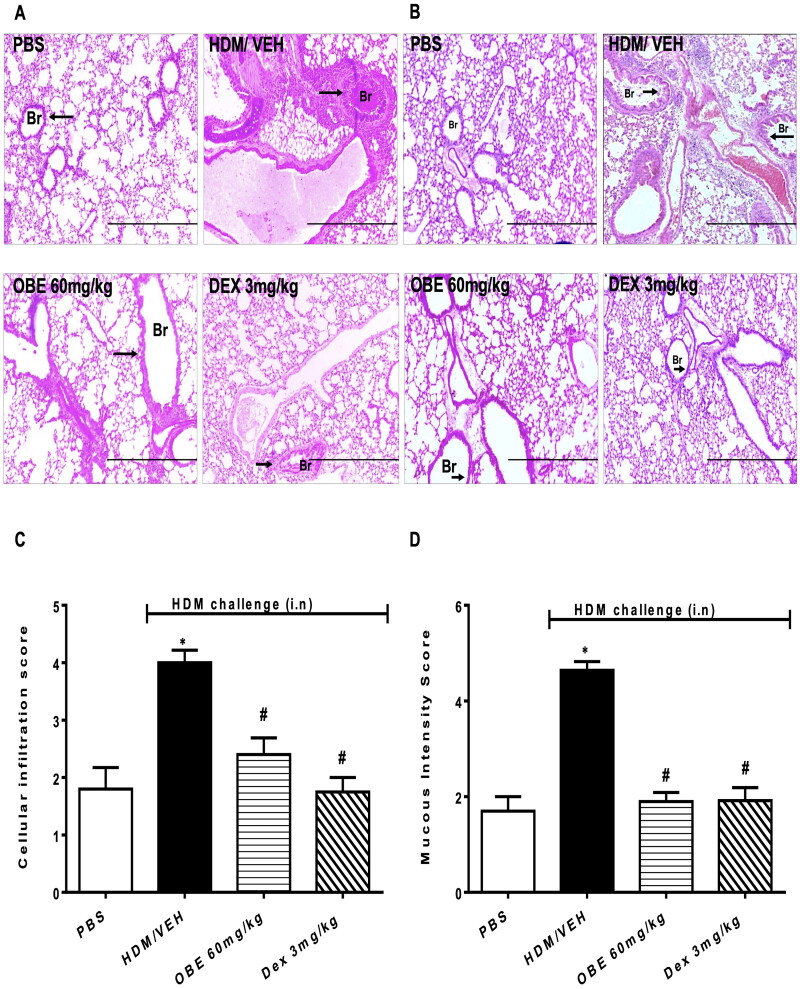
Effect of therapeutic OBE treatment, at a single dose of 60 mg/kg; i.p., on the HDM-induced airway inflammation and airway remodeling. Histopathological changes displaying (A) H&E and (B) PAS staining of lungs sections are shown as low-magnification light photomicrographs displaying the different treatment groups; PBS-challenged mice post-treated with vehicle (PBS group), HDM-challenged mice post-treated with vehicle (HDM group), HDM-challenged mice post-treated with OBE (60 mg/kg; i.p) (OBE group) and HDM-challenged mice post-treated with DEX (3 mg/kg; i.p) (DEX group); scale bar = 200 µm. Bar graph shows (C) cellular infiltration and (D) mucous intensity score for H&E and PAS staining, respectively. Data are expressed as mean ± SEM (*n* = 5–7). *p < 0.05 vs. PBS and #*p* < 0.05 vs. HDM (H&E- ANOVA followed by bonferroni *post hoc* test, PAS – Kruskal-Wallis test followed by Dunn’s multiple comparison test). Br = bronchioles. Arrows indicate peribronchial and perivascular inflammation (H&E) or significant bronchial mucus production and goblet cell hyper/metaplasia (PAS).

Histopathological assessment using H&E and PAS stains showed that lungs from HDM-challenged mice exhibited severe perivascular and peribronchial inflammatory cell infiltration (cellular infiltration score, HDM vs. PBS, 4.0 ± 0.2 vs. 1.8 ± 0.4, *p* < 0.05; Figure 4A, C, *n* = 5–7). Additionally, HDM challenge significantly enhanced goblet cell hyper/metaplasia and bronchial mucus production (mucous intensity score, HDM vs. PBS, 4.6 ± 0.2 vs. 1.7 ± 0.3, *p* < 0.05; Figure 4B, D, *n* = 5–7), thus clearly showing that the airway remodeling was established 3 days after the last HDM challenge. However, lung sections from the PBS group showed normal histology (*p* < 0.05; Figure 4A–D, *n* = 5–7). OBE (60 mg/kg) treatment significantly reversed HDM-induced histopathology; in the perivascular and peribronchial inflammatory cell infiltration (cellular infiltration score, OBE vs. HDM, 2.4 ± 0.3 vs. 4.0 ± 0.2; *p* < 0.05, Figure 4A, C) and the goblet cell hyper/metaplasia and bronchial mucus production (mucous intensity score, OBE vs. HDM, 1.9 ± 0.2 vs. 4.6 ± 0.2; *p* < 0.05, Figure 4B, D). Therapeutic OBE (60 mg/kg) treatment achieved similar histological appearance and score to those observed in the DEX treatment group (*p* < 0.05; Figure 4A, B).

## Effect of therapeutic OBE treatment on HDM- induced phosphorylation levels of EGFR, ERK1/2 and AKT

**Figure 5.**
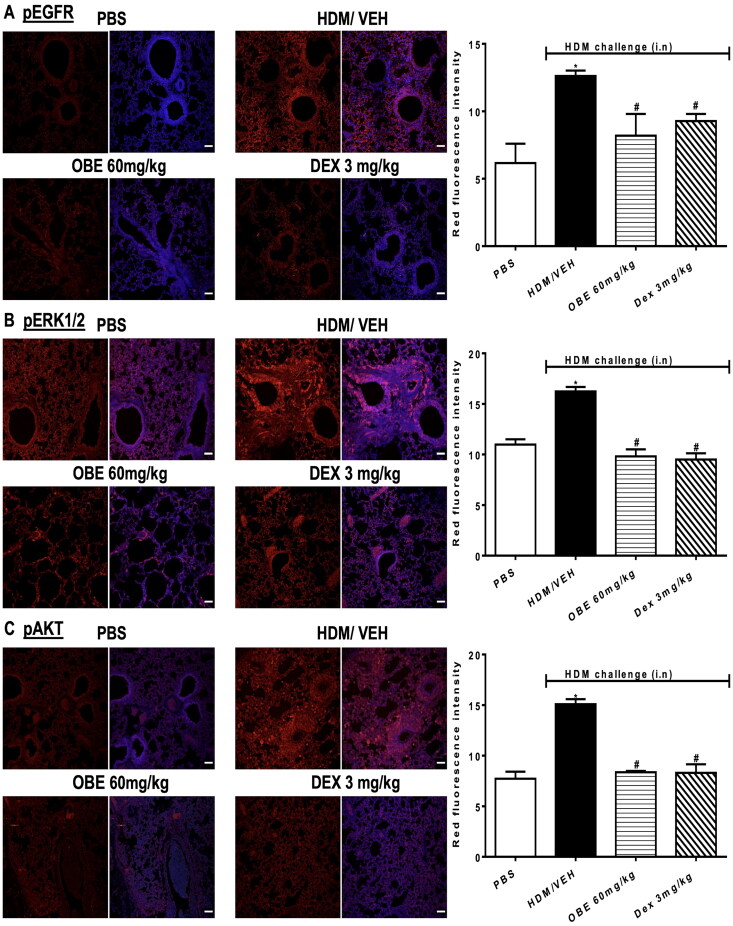
Effect of therapeutic OBE treatment, at a single dose of 60 mg/kg; i.p, on the HDM-induced expression of phosphorylated (A) EGFR (B) ERK1/2 and (C) AKT. Lung sections were obtained from the treatment groups; PBS-challenged mice post-treated with vehicle (PBS group), HDM-challenged mice post-treated with vehicle (HDM group), HDM-challenged mice post-treated with OBE (60 mg/kg; i.p) (OBE group) and HDM-challenged mice post-treated with DEX (3 mg/kg; i.p) (DEX group), thereafter these lung sections were immunostained for pEGFR, pERK1/2 and pAKT. The left-hand panel displays the expression of pEGFR, pERK1/2 and pAKT (red), whilst the right-hand panel displays the overlay with DAPI (blue), scale bar = 50 µm. Bar graphs represent the quantitative assessment of fluorescence intensity of pEGFR, pERK1/2 and pAKT (arbitrary units). Data are expressed as mean ± SEM (*n* = 4–5). *p < 0.05 vs. time-matched PBS-challenged mice, ^#^*p* < 0.05 vs. HDM-challenged mice (Kruskal-Wallis test followed by Dunn’s multiple comparison test).

Expression of pEGFR, pERK1/2 and pAKT was significantly increased following HDM challenge by 105.0, 40.0, and 95.3%, respectively, compared to PBS group, as determined by immunofluorescence intensity (*p* < 0.05; Figure 5A–C, *n* = 4–5). Treatment with OBE (60 mg/kg) substantially reversed the phosphorylation of EGFR (68.8%; *p* < 0.05), ERK1/2 (124.0%; *p* < 0.05) and AKT (91.1%; *p* < 0.05) induced by HDM-challenge and was comparable to the inhibitory effects exhibited by the DEX treated group (*p* < 0.05; Figure 5A–C, *n* = 4–5).

### Effect of preventative OBE treatment on HDM- induced inflammatory cell infiltration

**Figure 6.**
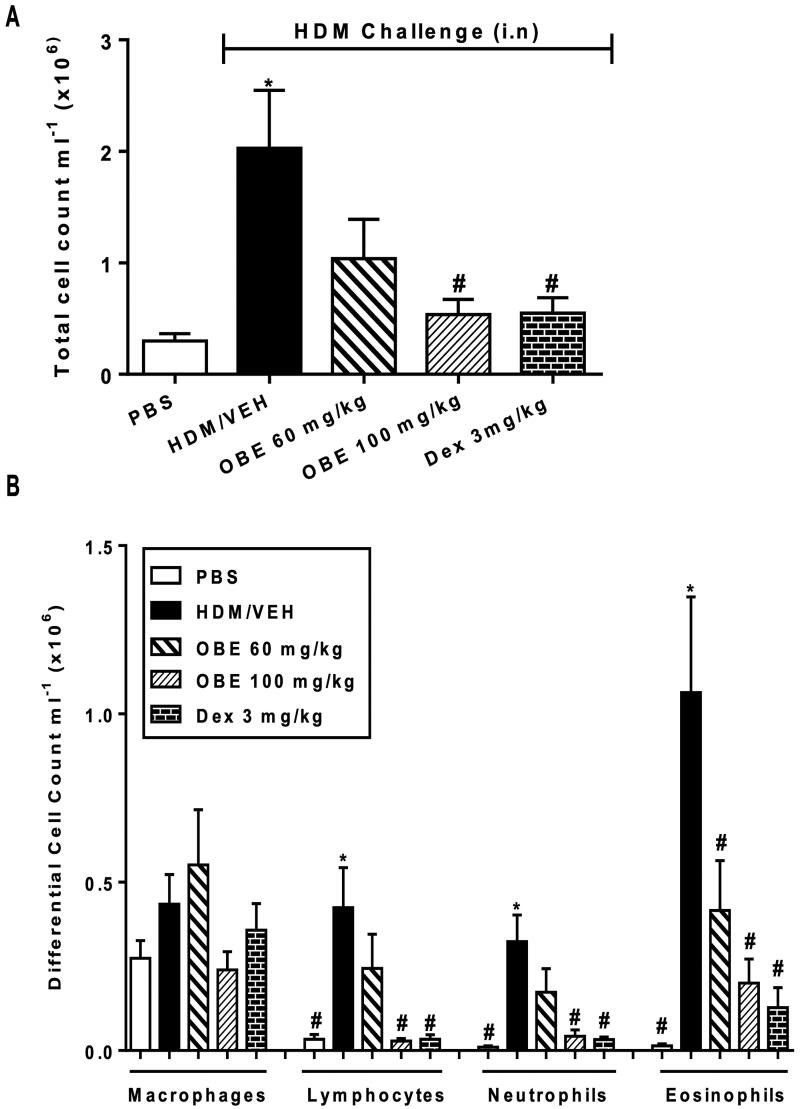
Effect of preventive treatment with OBE, at doses of 60 and 100 mg/kg; i.p., on the HDM-induced increase in (A) total cell and (B) differential cell count. Data are expressed as mean ± SEM (*n* = 8–11) *p < 0.05 vs. PBS and ^#^*p* < 0.05 vs. HDM (Kruskal-Wallis test followed by Dunn’s multiple comparison test).

HDM-challenged mice (VEH group), utilizing the preventative protocol, showed significantly elevated counts in total cell (2.0 ± 0.5 vs. 0.3 ± 0.06 × 10^6^ cells/mL), lymphocytes (0.4 ± 0.1 vs. 0.03 ± 0.01 cells × 10^6^ cells/mL; *p* < 0.05), neutrophils (0.3 ± 0.08 vs. 0.01 ± 0.003 cells × 10^5^ cells/mL; *p* < 0.05) and eosinophils (1.1 ± 0.3 vs. 0.01 ± 0.01 cells × 10^5^ cells/mL; *p* < 0.05; Figure 6A, B, *n* = 8–11). Preventive OBE (100 mg/kg) treatment significantly inhibited the increase in total cell and all the differential cell counts caused by the HDM challenge, whilst OBE (60 mg/kg) treatment significantly inhibited only the eosinophilia (*p* < 0.05; Figure 6A, B, *n* = 8–11). Moreover, inhibition of the cellular infiltration with OBE was comparable to that of DEX (Figure 6A, B).

### Effect of preventative OBE treatment on HDM-induced histopathological changes

**Figure 7.**
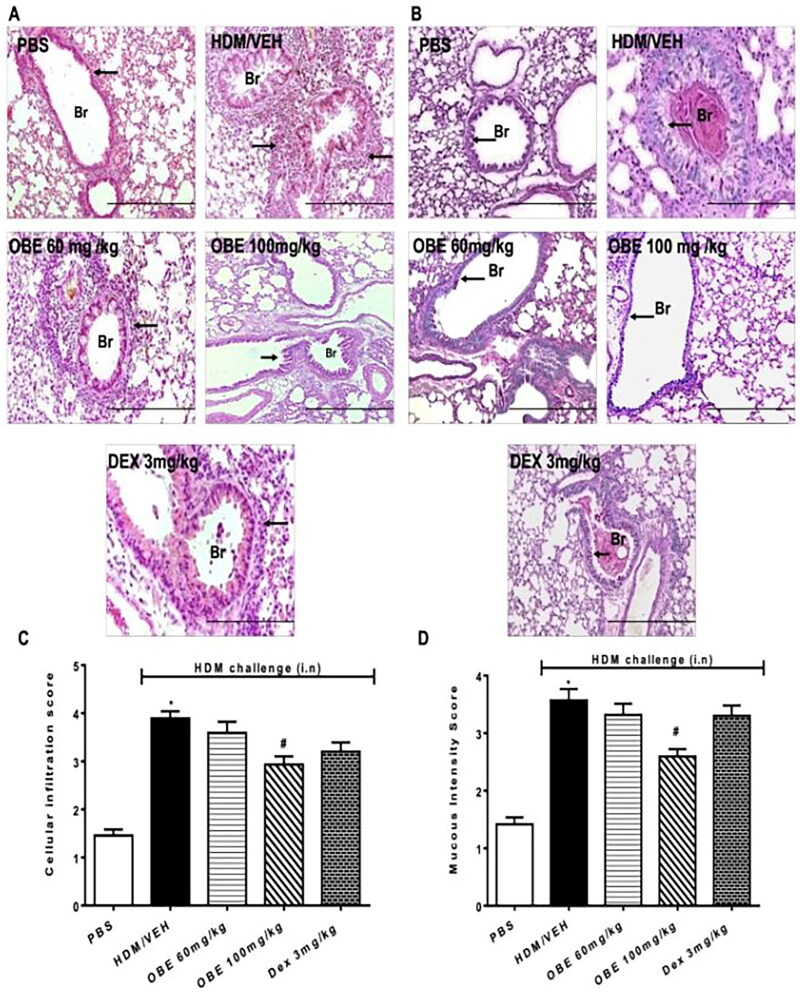
Effect of preventive treatment with OBE, at doses of 60 and 100 mg/kg; i.p., on the HDM-induced airway inflammation and airway remodeling. Histopathological changes displaying (A) H&E and (B) PAS staining of lungs sections from all treatment groups; PBS-challenged mice pre-treated with vehicle (PBS group), HDM-challenged mice pre-treated with vehicle (HDM group), HDM-challenged mice pre-treated with OBE (60 mg/kg; i.p) (OBE 60 group), HDM-challenged mice pre-treated with OBE (100 mg/kg; i.p.) (OBE 100 group) and HDM-challenged mice pre-treated with DEX (3 mg/kg; i.p.) (DEX group); scale bar = 200 µm. Bar graphs illustrate (c) cellular infiltration and (d) mucous intensity score for H&E and PAS staining, respectively. Data are expressed as mean ± SEM (*n* = 11-15). *p < 0.05 vs. PBS and ^#^*p* < 0.05 vs. HDM (H&E - ANOVA followed by bonferroni *post hoc* test, PAS - Kruskal-Wallis test followed by Dunn’s multiple comparison test). Br = bronchioles. Arrows indicate peribronchial and perivascular inflammation (H&E) or significant bronchial mucus production and goblet cell hyper/metaplasia (PAS).

Histological assessment using H&E and PAS stains showed, as noted previously, severe perivascular and peribronchial inflammatory cell infiltration in HDM-challenged lungs of mice (cellular infiltration score, HDM vs. PBS, 3.9 ± 0.1 vs. 1.5 ± 0.1, *p* < 0.05; Figure 7A, C) which resulted in notably enhanced goblet cell hyper/metaplasia and bronchial mucus production (mucous intensity score, HDM vs. PBS, 3.6 ± 0.2 vs. 1.4 ± 0.1, *p* < 0.05; Figure 7B, D). This HDM-induced airway remodeling was in-line with that observed in the therapeutic treatment protocol. Preventive OBE (60 mg/kg) treatment produced no significant effect on HDM-induced histopathological changes. However, preventive OBE (100 mg/kg) treatment resulted in a significant inhibitory effect on the HDM-induced: perivascular and peribronchial inflammatory cell infiltration (cellular infiltration score, OBE vs. HDM, 2.9 ± 0.2 vs. 3.9 ± 0.1, *p* < 0.05; Figure 7A, C, *n* = 11–15), goblet cell hyper/metaplasia and bronchial mucus production (mucous intensity score, OBE vs. HDM, 2.6 ± 0.1 vs. 3.6 ± 0.2, *p* < 0.05; Figure 7A, C). Of interest, treatment with DEX had no significant effect on perivascular and peribronchial inflammatory cell infiltration, goblet cell hyper/metaplasia and bronchial mucus production (*p* > 0.05; Figure 7A–D, *n* = 11–15).

### Effect of preventative OBE on HDM-specific IgE levels in sera

**Figure 8.**
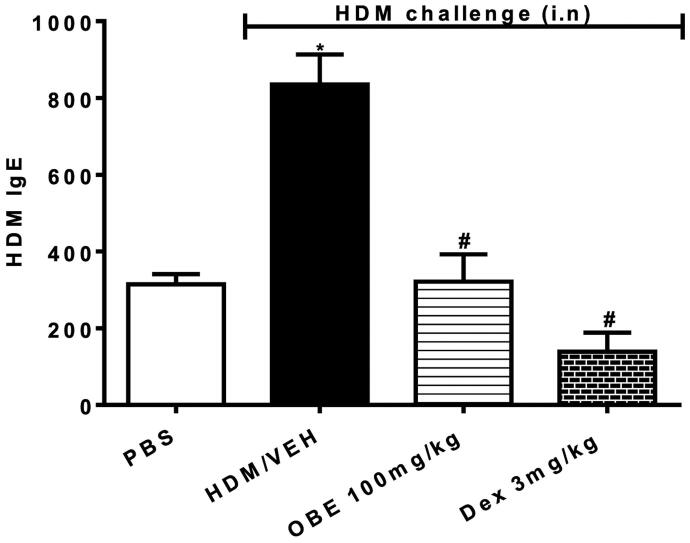
Effect of preventive treatment with OBE, at a single dose of 100 mg/kg; i.p., on the HDM-induced serum levels of HDM-specific IgE. The different treatment groups were; PBS-challenged mice pre-treated with vehicle (PBS group), HDM-challenged mice pre-treated with vehicle (HDM group), HDM-challenged mice pre-treated with OBE (100 mg/kg; i.p.) (OBE group) and HDM-challenged mice pre-treated with DEX (3 mg/kg; i.p.) (DEX group). Data are expressed as mean ± SEM (*n* = 6–11). *p < 0.05 PBS group, ^#^*p* < 0.05 vs. HDM group (Kruskal-Wallis test followed by Dunn’s multiple comparison test).

HDM-challenged mice (HDM group) showed substantially elevated levels of HDM-specific IgE in the sera in comparison to the PBS group (835.3 ± 78.0 vs. 314.7 ± 26.0; *p* < 0.05, Figure 8, *n* = 6–11). Preventive OBE (100 mg/kg) treatment substantially decreased the levels of HDM-specific IgE in sera compared to the HDM group (321.4 ± 71.4 vs. 835.3 ± 78.0; *p* < 0.05, Figure 8, *n* = 6–11). DEX treatment significantly reduced the HDM-specific IgE levels, (*p* < 0.05, Figure 8) to a greater extent than the OBE (100 mg/kg) treatment, however the difference between these two treatments was not significant.

### Effect of preventative OBE treatment on the levels of various cytokines in the airways

**Figure 9.**
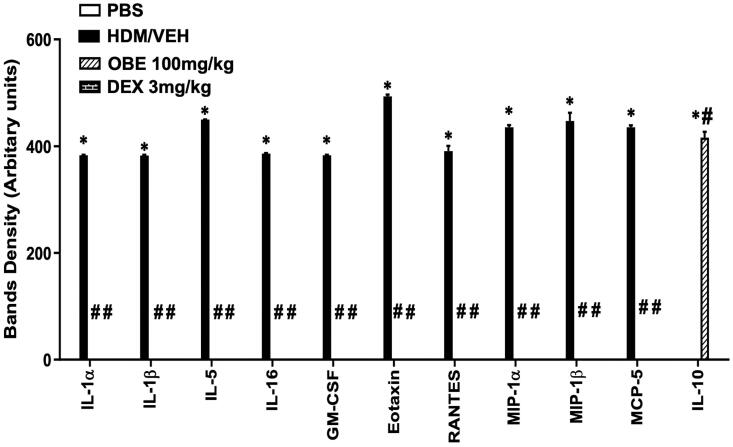
Effect of preventive treatment with OBE, at a single dose of 100 mg/kg; i.p, on the HDM-induced expression of various pro-inflammatory cytokines. Data are expressed as mean ± SEM (*n* = 3). *p < 0.05 vs. PBS group, ^#^*p* < 0.05 vs. HDM group (ANOVA followed by Bonferroni *post hoc* test).

HDM-challenged mice (HDM group) had significantly enhanced expression of numerous pro-inflammatory cytokines, which is seen in the asthma-like phenotype, such as IL-1α, IL-1β, IL-5, IL-16, GM-CSF, eotaxin, RANTES, MIP1α, MIP1β and MCP-5 (*p* < 0.05; Figure 9, *n* = 3) compared to the PBS group. In contrast, preventive OBE (100 mg/kg) treatment significantly suppressed the HDM-induced increased expression of these cytokines (*p* < 0.05; Figure 9, *n* = 3). However, IL-10 levels, an anti-inflammatory cytokine were significantly enhanced in the OBE group compared to the HDM group (*p* < 0.05; Figure 9, *n* = 3).

### Effect of preventative OBE treatment on HDM-induced phosphorylation levels of EGFR, ERK1/2 and AKT

**Figure 10.**
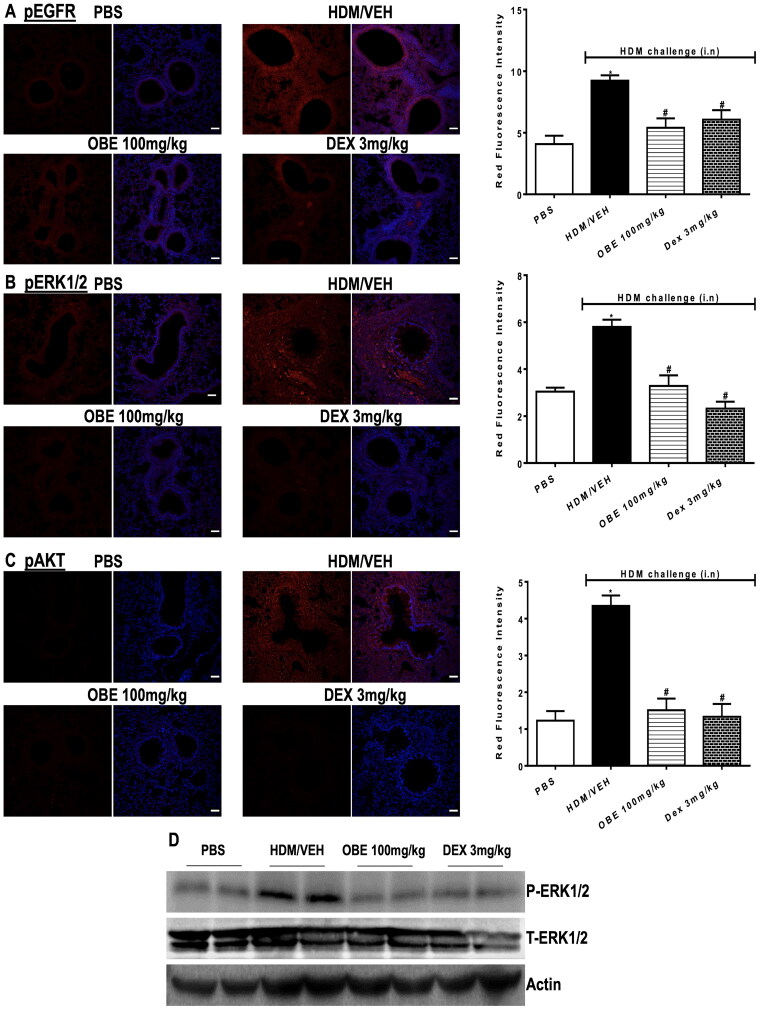
Effect of preventive treatment with OBE, at a single dose of 100 mg/kg; i.p., on the HDM-induced expression of phosphorylated (A) EGFR (B) ERK1/2 and (C) AKT. Lung sections were taken from the different treatment groups; PBS-challenged mice pre-treated with vehicle (PBS group), HDM-challenged mice pre-treated with vehicle (HDM group), HDM-challenged mice pre-treated with OBE (100 mg/kg; i.p) (OBE group) and HDM-challenged mice pre-treated with DEX (3 mg/kg; i.p.) (DEX group), thereafter these lung sections were immunostained for pEGFR, pERK1/2 and pAKT. The left-hand panel displays the expression of pEGFR, pERK1/2 and pAKT (red), whilst the right-hand panel displays the overlay with DAPI (blue); scale bar = 50 µm. Bar graphs presents the quantitative assessment of fluorescence intensity of pEGFR, pERK1/2 and pAKT (arbitrary units). Data are expressed as mean ± SEM (*n* = 4–5). *p < 0.05 vs. time-matched PBS-challenged mice, ^#^*p* < 0.05 vs. HDM-challenged mice (Kruskal-Wallis test followed by Dunn’s multiple comparison test). Western blot analysis of (D) pERK1/2 and total ERK1/2 protein levels from lungs with different treatment groups. The blots are of two pooled lung sample (*n* = 6, total).

HDM-challenged mice (HDM group) resulted in a significantly elevated expression of pEGFR, pERK1/2 and pAKT by 125.5, 90.7, and 255.7%, respectively, in contrast to the PBS group, as detected by immunofluorescence intensity (*p* < 0.05; Figure 10A–C, *n* = 4–5). Preventive OBE treatment (100 mg/kg) significantly suppressed the HDM-induced phosphorylation of EGFR (74.4%; *p* < 0.05), ERK1/2 (91.3%; *p* < 0.05) and AKT (91.0%; *p* < 0.05) which was similar to the inhibitory effect identified in DEX treated group (*p* < 0.05, Figure 10A–C, *n* = 4–5).

To validate the immunofluorescence data, Western blot was utilized, thus providing an approach to assess the expression of pERK1/2. Western blot analysis of lung homogenate has shown that HDM group produced an elevated level of pERK1/2 (but not T-ERK1/2) compared to the PBS group (Figure 10D). Preventive OBE (100 mg/kg) treatment produced a noticeable inhibitory effect on pERK1/2 level which was comparable to the DEX group. Therefore, the Western blot confirmed the increased p-ERK1/2 levels observed in immunofluorescence was correct (*n* = 6, total for each group).

## Discussion

The major findings of our study reveal that OBE reversed established allergic inflammation, both in terms of cytological and histopathological changes, and activation of the signaling molecules EGFR, ERK1/2 and AKT. Furthermore, our data also shows that treatment with OBE, prior to immunization with HDM, significantly reduced the HDM-induced increased BAL cellularity, histopathological changes, the upregulation of EGFR, ERK1/2 and AKT expression, cytokine release and also HDM-specific IgE. The findings of this study suggest that treatment with OBE not only reverses asthma-like features but can also inhibit their development when administered prior to immunization.

A significant number of preclinical studies have shown that herbs and other natural products possess potent, effective and diverse pharmacological actions thus justifying their medicinal use (Li et al. 2015; Rehman et al. 2019; Cheung et al. 2020). Emerging clinical studies have also provided evidence that natural products can reduce disease features, in several disease states, as well as reduce the risk of developing some diseases (Zhang et al. 2016; Nile and Kai 2021).

Asthma models are valuable tools to investigate airway diseases in the setting of an intact immune system and have been used for decades (Epstein 2006). However, their usefulness has been questioned recently on the basis that drugs with demonstrated efficacy in animal models of asthma, showed little clinical benefit in humans (Martin et al. 2014). An important factor in determining clinical relevance of efficacy of a preclinical drug, is the timing of drug dosing. For example, asthma is an on-going chronic inflammatory condition and therefore it is particularly important that any successful asthma therapy is able to show efficacy in reversing established airway remodeling since any novel drug will be administered to patients with established airway disease (Martin et al. 2014).

We previously reported that OBE reduces many inflammatory indices in an HDM-induced murine model of allergic inflammation (El-Hashim et al. 2020). However, in that study, OBE was administered 1 h prior to the allergen nasal challenge and whilst it provided clear ‘‘proof of concept” that OBE induced effective anti-inflammatory actions, the prophylactic nature of the treatment protocol, coupled with the close timing of the OBE administration to the allergen challenge, do not simulate the clinical scenario as closely as required. Indeed, in clinical practice, treatment is normally initiated in response to an established disease and not before. Therefore, for OBE, or any of its fractions or active constituents, to be incorporated in any clinical treatment protocol, it has to demonstrate efficacy in reversing established asthma-phenotype in clinically relevant animal models. Collectively, our data shows that therapeutic treatment with OBE reversed the HDM-induced airway cellularity and histopathological changes. The effect was significant at the 60 mg/kg (OBE) dose, similar to what we had previously noted in the prophylactic treatment study (El-Hashim et al. 2020) and was equally as effective as the steroid treatment. This is quite remarkable, as reversing or downregulating inflammatory changes once established is not easily achieved with anti-inflammatory therapy, or even with steroid therapy (Malmstrom et al. 1999; Zhang WX and Li 2011). Therefore, the ability of OBE to reverse inflammation is noteworthy and certainly an important requirement for any potential adjunct therapy for asthma, or any other inflammatory based disease.

The exact mechanisms by which OBE reverses airway inflammation remains to be established. However, we and others have previously identified the EGFR, ERK1/2 and AKT to be an important signaling pathway in the development of pathophysiological changes in the murine allergic asthma model and is indeed susceptible to inhibition by OBE treatment (Takeyama et al. 2001; El-Hashim et al. 2017, 2020). In this study, we also note that HDM challenge resulted in activation of EGFR, ERK1/2 and AKT which persisted for several days after allergen challenge. It is likely that the activation of the EGFR, ERK1/2 and AKT signaling pathway was, at least partly, responsible for the HDM-induced inflammatory changes. The activation of EGFR, ERK1/2 and AKT was suppressed by therapeutic OBE treatment, and this was as effective as the DEX treatment. Whilst our data suggest that the EGFR, ERK1/2 and AKT signaling pathway could be mainly responsible for the anti-inflammatory effects of OBE, other studies have shown that OBE has numerous other anti-inflammatory actions, such as inhibition of MAPK family, mammalian target of rapamycin, tissue inhibitors of metalloproteinases, interferon-γ, oxidative stress, pro-inflammatory enzymes such as cyclooxygenase 2, and various C-C and C-X-C chemokines (Marefati et al. 2018; Khajah et al. 2019; El-Hashim et al. 2020). While many of the potential asthma drugs that selectively target single mediators have shown positive effects in preclinical animal models, when administered prophylactically, their effectiveness was not mirrored in clinical trials (Yamashita et al. 2002; Molfino et al. 2016) and the drugs that did make it to clinical practice, have limited impact and/or use in the management of asthma (Agache et al. 2020). The reasons for this are unclear but could be partly due to the overlapping actions of many inflammatory mediators resulting in some degree of redundancy (Mantovani 2018). Therefore, the board based anti-inflammatory actions of OBE may be a key to its ability to inhibit the asthma phenotype. Moreover, it is most likely that the anti-inflammatory effects observed with OBE are due to the many chemical constituents present in it.

Our data showed that OBE also had a remarkable effect on eosinophil resolution. Eosinophils are primary cells thought to drive airway damage in asthma and make up a significant number of cells in the airways of asthmatic patients and allergen-challenged allergic animals (El-Hashim et al. 2020; Esmaeilzadeh et al. 2021). We have previously shown that pretreatment with OBE significantly inhibited the recruitment of eosinophils in the airway of HDM-challenged mice, most likely by inhibiting the release of pro-inflammatory mediators since it also decreased chemotaxis of eosinophils *in vitro* (El-Hashim et al. 2020). Whilst it is likely that treatment with OBE prevented further eosinophil recruitment into the airways, it is more plausible that OBE treatment promoted eosinophil apoptosis which resulted in the reduced eosinophilia at day 21. This assertion is supported by our previous findings that OBE induces neutrophil apoptosis (Khajah et al. 2019). Therefore, it is possible that OBE reverses inflammation, at least partly; *via* resolution of inflammation.

Focus has recently shifted on emerging evidence showing that a diet can have strong effects on health and disease. Some evidence suggests that a good diet, which is rich in antioxidants sources (e.g., fruits and vegetables) such as the Mediterranean diet may be beneficial in the primary prevention of many diseases including asthma. Based on the anti-asthma properties of onion, demonstrated in both clinical (Clark et al. 2010) and preclinical studies (Dorsch et al. 1990; El-Hashim et al. 2020), we investigated if administration of OBE, prior to the immunization process, would affect the allergic response and/or allergic airway inflammation. Our findings show that preventative treatment with OBE dose-dependently inhibited the total and BAL cellularity and significantly inhibited the HDM-induced perivascular and peribronchial inflammation and goblet cell hyper/metaplasia. However, the effect of DEX on the histopathological changes was not significant. The lack of effect of DEX on the airway remodeling could be that this change was more resistant to steroid inhibition. Alternatively, higher doses or longer duration of treatment were needed to result in significant inhibition of the histological changes. It is of interest to note that there were two main differences between the preventative and therapeutic treatment protocols. The first being that the 60 mg/kg dose produced significant effects in the therapeutic treatment regimen, in both cellular and histopathological parameters, but had minimal effects in the preventative regimen, and inhibition was mainly achieved with a higher dose of 100 mg/kg. The second difference is that even with the higher dose (100 mg/kg), the degree of inhibition in the histopathological changes was notably less in comparison with the 60 mg/kg in the therapeutic treatment protocol. It remains to be determined whether continuing the OBE treatment for a longer period would result in greater inhibition of the histopathological changes. A similar preventative treatment approach also produced an anti-inflammatory action in colitis model of inflammation and was also less effective than the therapeutic treatment approach (Khajah et al. 2020). Our findings are in line with studies showing that a diet rich in fruits and/or vegetables is associated with reduction in indices of inflammation (Li et al. 2015; Grosse et al. 2020). Furthermore, clinical studies have shown ‘protective’ associations for fruits, vegetables, and fish intake in childhood asthma (Garcia-Larsen et al. 2018; Papamichael et al. 2018).

To investigate the mechanisms by which the preventative OBE treatment regimen may have reduced the HDM-induced asthma phenotype, we assessed its effects on HDM-specific IgE, an important outcome of sensitization. Our data show that OBE treatment significantly inhibited the development of HDM-specific IgE. This finding is in agreement with studies showing that inhibition of the immunization process inhibits IgE, and the IgE-dependent-allergic inflammatory response to allergen challenge (Mishra et al. 2015). Therefore, the inhibitory effect of OBE on IgE levels could, at least partly, explain the mechanism by which OBE inhibits the development of the allergic inflammatory phenotypes. The mechanisms by which OBE may reduce HDM-specific IgE are not known. However, one could speculate that this may be *via* inhibition of cytokines that regulate the complex process of the sensitization, possibly by interfering with the allergen presentation stage, differentiation of Th0 cells into Th2 cells, maturation/inhibition of B cells, etc.

To further elucidate the effects of early treatment with OBE on the asthma phenotype, we investigated its effect on a number of diverse pro-inflammatory molecules with different roles in asthma such as IL-1α and β, IL-5, IL-16, the colony-stimulating factors, GM-CSF, the chemokines eotaxin, RANTES, MIP-1α, MIP-1β, MCP-5 and also the anti-inflammatory IL-10. Our findings show that the preventative OBE treatment protocol resulted in a significant reduction in all of these molecules, with a degree of inhibition that is comparable to that seen following treatment with standard anti-allergic/anti-asthma drugs (Seo et al. 2019). Of particular interest is the IL-5 cytokine which is an important cytokine for eosinophil differentiation, proliferation, recruitment, priming and activation and a target for several novel monoclonal antibodies in asthma therapy (Nagase et al. 2020). Our data indicate an association between IL-5 levels and eosinophils number in the airways, and that OBE showed effective inhibition on both IL-5 and eosinophils. In contrast, OBE treatment significantly increased the levels of the Th2 anti-inflammatory cytokine IL-10. Therefore, it is possible that the up-regulation of IL-10 levels may be one of the anti-inflammatory mechanisms of action of OBE. It is of interest to note that OBE’s inhibitory effects on cytokines and chemokines are in line with its effects on the HDM-specific IgE.

Although the molecular mechanisms by which OBE produces its anti-inflammatory action are not clear, our data suggests that it likely involves inhibiting the EGFR, ERK1/2 and AKT signaling pathway, which is an important pathway in the asthma phenotype. Therefore, collectively our findings show that treatment with OBE, whether given just before nasal HDM challenge (El-Hashim et al. 2020) or after HDM challenge or even before immunization with HDM, all result in modulation of the EGFR, ERK1/2 and AKT pathways. This suggests that OBE has powerful modulatory effect on this important signaling pathway. Nonetheless, in the preventative treatment protocol, the downregulation of the activation EGFR, ERK1/2 and AKT is likely due to an early reduction in the HDM-specific IgE as well as IL-5, amongst other inflammatory mediators, as a consequence of inhibition of the immunization process, rather than a direct effect on inflammation.

## Conclusions

Our data clearly show that treatment with OBE both reverses the asthma phenotype, once established, and reduces its severity when administered prior to the immunization and this occurs *via* inhibition of several mechanisms such as IgE, IgE-dependent allergic inflammation, numerous cytokines and chemokines and the EGFR, ERK1/2 and AKT signaling pathways. We believe that our data suggest that the intake of onion/OBE by asthmatic patients, in addition to their regular asthma medication, may offer some therapeutic benefit due to their anti-inflammatory actions and may even reduce the risk of developing IgE-mediated allergic disorders. However, appropriate clinical studies are needed to investigate this further.
